# Application of physiologically-based pharmacokinetic/pharmacodynamic models to evaluate the interaction between nifedipine and apatinib

**DOI:** 10.3389/fphar.2022.970539

**Published:** 2022-08-26

**Authors:** Hongrui Liu, Yiqun Yu, Lu Liu, Chunyan Wang, Nan Guo, Xiaojuan Wang, Xiaoqiang Xiang, Bing Han

**Affiliations:** ^1^ Department of Pharmacy, Minhang Hospital, Fudan University, Shanghai, China; ^2^ Department of Clinical Pharmacy, School of Pharmacy, Fudan University, Shanghai, China

**Keywords:** pharmacodynamics, drug-drug interaction (DDI), physiologically-based pharmacokinetics (PBPK), nifedipine, apatinib

## Abstract

**Aim:** It has been found that the co-administration of nifedipine with apatinib could cause exposure changes of nifedipine *in vivo*. But, whether this pharmacokinetic drug-drug interaction (DDI) between nifedipine and apatinib could enhance the antihypertensive effect of nifedipine, causing sever changes of blood pressure was unknown. Therefore, the aim of the present study was to conduct the pharmacokinetic/pharmacodynamic (PK/PD) modelling to evaluate the effect of pharmacokinetic changes on the antihypertensive effect of nifedipine. Thus, the results could guide the co-administration of these two drugs in clinic.

**Methods:** A physiologically-based pharmacokinetic (PBPK) model was first developed for nifedipine. The pharmacokinetic DDI between nifedipine and apatinib was evaluated. Then the verified PBPK models were linked to a PD model for investigating whether the exposure changes of nifedipine could cause severe changes in blood pressure. Furthermore, the changes in blood pressure caused by combination with apatinib were also assessed in patients with hepatic impairment *via* the PBPK/PD models.

**Results:** The predicted area under plasma concentration-time profile (AUC), maximum concentration (C_max_), area under effect-time profile (AUE), and maximum reduction in systolic blood pressure (R_max_) are all within 0.5–2.0-fold of the observed data, indicating that the PBPK/PD models for nifedipine are successfully established. The increases of predicted AUC and C_max_ of nifedipine in the presence of apatinib are 1.73 and 1.41-fold, respectively. Co-administration of nifedipine with apatinib could cause exposure changes of nifedipine *in vivo*. However, the predicted AUE and R_max_ changes of nifedipine in the presence to the absence of apatinib in cancer patients as well as in patients with hepatic impairment are all within 1.25-fold. The results indicate that the exposure changes of nifedipine caused by combination of apatinib has little effect on the changes of systolic blood pressure both in cancer patients and patients with hepatic impairment.

**Conclusion:** The pharmacokinetic changes of nifedipine caused by co-administration with apatinib has little impact on the antihypertensive effect of nifedipine. Apatinib is unlikely to cause severe pharmacodynamic DDI *via* inhibition of CYP3A4. It is suggested that nifedipine could be used in combination with apatinib without dose adjustment in clinic.

## Introduction

Apatinib, a small-molecule tyrosine kinase inhibitor, suppresses the tumor angiogenesis by selectively inhibiting the activity of vascular epidermal growth factor receptor (VEGFR) tyrosine kinases (Tian et al., 2011; Qiu et al., 2018; Meng et al., 2020). Apatinib was approved by the National Medical Products Administration (NMPA) for patients suffering from advanced gastric adenocarcinoma or gastric esophageal junction cancer in 2014 (Roviello et al., 2016; Geng et al., 2018). In 2020, the new indications for apatinib were approved for the treatment of advanced hepatocellular carcinoma. It has been reported that apatinib showed certain benefit in the treatment of other malignant tumors, such as non-small cell lung cancer, breast cancer, and colorectal cancer (Zhang, 2015; Xue et al., 2018; Maroufi et al., 2020). Although the clinical efficacy of apatinib is agreeable, further research on the potential drug–drug interactions (DDIs) is yet needed since cancer patients usually receive combination therapy, many of which are enzymes inhibitors or inducers. It is of great significance to evaluate the potential DDI risks associated with apatinib for its reasonable administration in clinic.

The *in vitro* metabolism studies showed that apatinib was mainly metabolized *via* CYP3A4/5, and CYP2D6 (Ding et al., 2013). Research has shown that the area under plasma concentration-time profile (AUC) of apatinib was significantly affected by co-administration with itraconazole (AUC_0-t_ increased by 75%) or rifampin (AUC_0-t_ decreased by 83%) in humans (Liu et al., 2018). In our previous study, the DDI simulation showed 2 to 4-fold changes in apatinib exposures by moderate CYP3A4 inhibitors and CYP3A4 inducers (Liu et al., 2021). A moderate increase of apatinib exposure (1.25 to 2-fold) was found with strong CYP2D6 inhibitor (Liu et al., 2021). However, the detailed evaluation of apatinib as a perpetrator in CYP450-based DDIs is lacking. Apatinib was reported to exert potent inhibition on CYP3A4 and CYP2C9 with the IC_50_ values of 1.80, 0.83, and 0.44 μM for midazolam hydroxylation, testosterone hydroxylation, and tolbutamide hydroxylation, respectively (Zhu et al., 2020). Thus, apatinib might affect the plasma exposures of CYP3A4 and CYP2C9 substrates when using combination therapy causing clinical efficacy and safety issues.

Many cancer patients were also treated with antihypertensive agents concomitantly. The combination of calcium channel blockers (CCB) was unavoidable, among which nifedipine was one of the most commonly used due to the rapid onset of action without central nervous system depression (Haddad, 1996; Ndanusa et al., 1997). Besides, hypertension is a commonly reported adverse event for patients receiving apatinib and, therefore, co-administration of nifedipine with apatinib was common in clinical practice (Peng et al., 2018; Shao et al., 2020). The *in vitro* studies indicated that nifedipine was a typical substrate of CYP3A4 which was almost completely metabolized by CYP3A4 (Guengerich et al., 1986). The research conducted by Zhu et al. (2020) has confirmed that co-administration of nifedipine with apatinib significantly increased the AUC_0–48h_ and C_max_ of nifedipine by 83% and 64%, respectively. Co-administration of apatinib with nifedipine could cause exposure changes of nifedipine *in vivo*. But, whether this pharmacokinetic DDI might cause significant clinical effects, in other words, whether enhancing the antihypertensive effect of nifedipine leading to a risk of hypotension is still unknown (Welch and Todd, 1990). The dosing regimen of nifedipine when co-administrated with apatinib might be of vital importance. That is to say, when apatinib and nifedipine are co-administered, should the nifedipine be administered in regular dose in clinic, or the dose needs to be reduced. If the dose of nifedipine needs to be reduced, what is the appropriate dose?

In the present study, physiologically-based pharmacokinetic (PBPK) modelling was applied to evaluate the impact of apatinib on the exposure of nifedipine. Meanwhile, the developed PBPK models were linked to a pharmacodynamic (PD) model to investigate whether the exposure changes of nifedipine could cause severe changes in blood pressure (Chetty et al., 2014). Furthermore, the DDIs between nifedipine and apatinib were also assessed *via* the PBPK/PD models to determine the reasonable combination regimen in patients with hepatic impairment. The results of the present study could clarify the potential DDI risks *via* inhibition on CYP3A4 by apatinib, providing data basis for guiding the reasonable application in clinic.

## Materials and methods

### Development and verification of nifedipine physiologically-based pharmacokinetic model

The PBPK model for nifedipine was built-in with Simcyp™ (version 16, Simcyp Limited, Sheffield, United Kingdom), a commercially available PBPK software. Nifedipine has low solubility and high permeability, which belongs to the Biopharmaceutics Classification System Class II drug (Nader et al., 2016). CYP3A4 metabolism is the predominantly eliminated pathway (Guengerich et al., 1986; Nader et al., 2016). The input parameters for nifedipine are summarized in Table 1. The physicochemical parameters such as molecular weight, oil/water partition coefficient (log P), dissociation equilibrium constants (pK_a_), plasma unbound fraction (f_u_), and blood to plasma partition coefficient (B/P) were obtained from the nifedipine compound file in Simcyp. The *in vivo* absorption of nifedipine controlled release (CR) tablet was described using the advanced dissolution, absorption, and metabolism (ADAM) model. The effective permeability (P_eff_) of nifedipine in human was calculated using the permeability data of MDCK II (Polli et al., 2001). The *in vivo* release of nifedipine CR tablet was described by Weibull function of dissolution profiles (Doki et al., 2017). The first-order model was used to describe the *in vivo* absorption of nifedipine immediate release (IR) tablet. A minimal PBPK distribution model and enzyme kinetics with recombinant CYP enzyme data were used to characterize the elimination of nifedipine in the Simcyp.

**TABLE 1:** Summary of input data for nifedipine in Simcyp™.

Parameters		Value	Source
Physiochemical parameters			
Molecular weight (g/mol)		346.3	Simcyp built-in data
log P		2.69	
Compound type		Monoprotic base	
pKa		2.82	
B/P		0.685	
f_u_		0.039	
Absorption parameters			
First-order model for IR tablet	*f*_a_	1	Simcyp built-in data
	k_a_ (1/h)	4.6	
ADAM model for CR tablet	MDCK II permeability (10^−6^ cm/s)	61	Polli et al. (2001)
	P_eff_ (10^−4^ cm/s)	10.5	Simcyp calculated
Disposition parameters			
Minimal PBPK model		0.57	Simcyp calculated
V_ss_ (L/kg)			
Elimination parameters			
Enzyme		rCYP3A4	Simcyp built-in data
V_max_ (pmol/min/pmol CYP)		22	
K_m_ (μM)		10.95	
Enzyme		rCYP3A5	
V_max_ (pmol/min/pmol CYP)		3.5	
K_m_ (μM)		31.9	

The developed PBPK models for nifedipine were verified with reported clinical study on a single-dose of 60 mg nifedipine CR tablet or 20 mg nifedipine IR tablet in healthy volunteers (Holtbecker et al., 1996; Toal et al., 2012). A Sim-Healthy volunteer population with population size of 100 in Simcyp at the age of 26–65 years old were used for the verification. The proportion of female was 0.5. The predicted results were compared with the clinical observations, and the predicted accuracy was measured by calculating the fold error of the pharmacokinetic parameters (C_max_, T_max_, and AUC), as described in Eq. 1 (Zhang et al., 2018; Li et al., 2019; Li et al., 2021).

$$\mathrm{fold}\;\mathrm{error}=\frac{\text{simulated}}{\text{observed}}$$ (1)

### Development of nifedipine-apatinib drug-drug interaction model

The PBPK models for apatinib were already developed and verified in the previous study (Liu et al., 2021). The predicted mean plasma concentration-time curves of apatinib in the presence or absence of itraconazole and rifampin were matched well with the clinical observed ones. Summary of the input data are listed in Table 2.

**TABLE 2:** PBPK model parameters for apatinib (Liu et al., 2021).

Parameters		Value	Unit
Physiochemical parameters			
Molecular weight		397.48	g/mol
log P		3.14	
Compound type		Diprotic base	
pKa		pKa_1_ = 6.60 pKa_2_ = 5.31	
B/P		0.995	
f_u_		0.076	
Absorption parameters			
ADAM model	Caco-2 permeability	6.81 × 10^−6^	cm/s
	P_eff_	0.80 × 10^−4^	cm/s
Disposition parameters			
Full PBPK model			Poulin and theil method
K_p_ scalar		0.7	
V_ss_ (L/kg)		2.684	
Elimination parameters			
CYP2D6			
V_max_		9.82	pmol/min/mg protein
K_m_		1.41	μM
CYP3A4			
V_max_		39.1	pmol/min/mg protein
K_m_		2.18	μM
CYP3A5			
V_max_		3.28	pmol/min/mg protein
K_m_		1.93	μM

Apatinib was reported to exert potent inhibition on CYP3A4 in a competitive way (Zhu et al., 2020). In the DDI models between nifedipine and apatinib, the K_i_ was set to 0.12, with the f_umic_ at 0.65 (Bao et al., 2018; Zhu et al., 2020). The PK profiles of nifedipine in the presence of apatinib were predicted and compared with the clinical observations. The subject characteristics as well as trial design were consistent with the reported single-dose DDI studies (Zhu et al., 2020). A Sim-Chinese volunteer population with population size of 24 in Simcyp at the age of 26–65 years old were used for the verification. The proportion of female was 0.5. Subjects either received a 30 mg of oral nifedipine CR tablet on day 1 or treated with 750 mg of oral apatinib once daily for eight consecutive days with concomitantly administration of 30 mg nifedipine CR tablet on day 6. The simulated results were compared with the observations (Zhu et al., 2020). The change of nifedipine exposure was determined by AUC ratio and C_max_ ratio, as described in Eq. 2 (Zhang et al., 2018; Li et al., 2019).

$$\text{AUC ratio}=\frac{\text{AUC with inhibitor}}{\text{AUC without inhibitor}}$$

or

$${\text{C}}_{\text{max}}\text{ ratio}=\frac{{\text{C}}_{\text{max}}\text{ with inhibitor}}{{\text{C}}_{\text{max}}\text{ without inhibitor}}$$ (2)

### Development and verification of pharmacodynamic model for nifedipine

Studies have shown that the control of systolic blood pressure (SBP) is more important than diastolic blood pressure (DBP) in most patients with hypertension (Donnelly et al., 1994; Meredith et al., 1994). Hence, SBP was employed in the PD modelling. An ordinary E_max_ model (Eq. 3) was used to describe the relationship between nifedipine concentration and the change of SBP (Abernethy et al., 1990; Donnelly et al., 1993). The E_max_ and EC_50_ were obtained from literature, which were set at −30 mmHg and 12.12 ng/ml, respectively (Edgar et al., 1987; Donnelly et al., 1994; Levine et al., 2003). The developed PD model was then linked to the PBPK model to investigate the effect of pharmacokinetic DDI between nifedipine and apatinib on the change of SBP. The predictive accuracy was measured by calculating the maximum reduction in SBP (R_max_) and the area under the effect-time curve (AUE) (Levine et al., 2003; Mourad, 2008; Toal et al., 2012).

$$\text{E}=\frac{E_{max}C}{EC_{50}+C}$$ (3)

### Application of the nifedipine physiologically-based pharmacokinetic/pharmacodynamic model

The developed PBPK/PD model was used to predict the changes in SBP caused by the exposure changes of nifedipine with the combination of apatinib. The virtual populations in Simcyp with sizes of 100 at the age of 26–65 years old were used in the simulations. The proportion of female was 0.5. Two clinical scenarios were simulated. The first scenario was designed to determine whether co-administration of apatinib with regular dose level of nifedipine could cause severe changes of SBP in cancer patients. The second scenario was designed to determine if dose adjustment of nifedipine was necessary in the combination of apatinib in patients with hepatic impairment.

## Results

### Verification of the physiologically-based pharmacokinetic model for nifedipine

**FIGURE 1:**
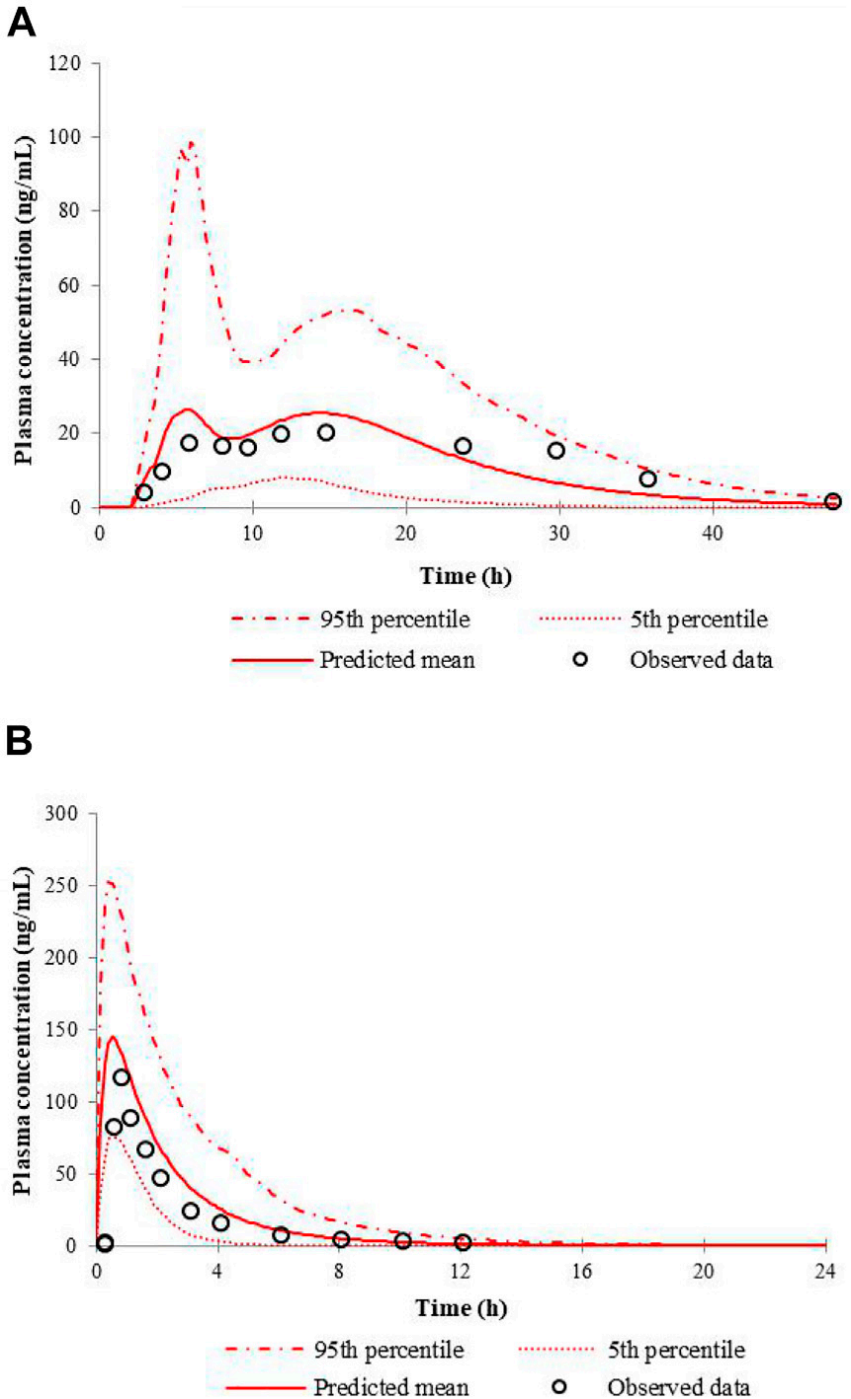
Predicted and observed mean plasma concentration-time curves of nifedipine following a single administration of 60 mg nifedipine CR tablet **(A)** or 20 mg nifedipine IR tablet **(B)** in healthy volunteers.

The simulated and the observed mean plasma concentration-time curves of nifedipine CR tablet at 60 mg or IR tablet at 20 mg dose levels in healthy volunteers are depicted in Figure 1. Table 3 shows the comparison of the model predicted pharmacokinetic parameters (C_max_, T_max_, and AUC) to the observed data, and the calculated fold errors of C_max_, T_max_, and AUC of nifedipine. The predicted mean plasma concentration-time curves of nifedipine were generally consistent with the clinical observed ones. Additionally, as shown in Table 3, the C_max_ and AUC values were successfully predicted within 2-fold of observed values. The results indicate a good prediction of the nifedipine PBPK model.

**TABLE 3:** Comparison of model-predicted C_max_, T_max_, and AUC to the observed data of nifedipine tablet.

Dose level	Parameters	Unit	Predicted	Observed	Fold error
60 mg nifedipine CR tablet	T_max_	h	5.76	4.99	1.15
	C_max_	ng/mL	26.51	22.00	1.20
	AUC	ng/mL·h	550	560	0.98
20 mg nifedipine IR tablet	T_max_	h	0.53	0.85	0.62
	C_max_	ng/mL	144.59	117.41	1.23
	AUC	ng/mL·h	369	230	1.60

### Evaluation of the drug–drug interactions prediction for nifedipine and apatinib

**FIGURE 2:**
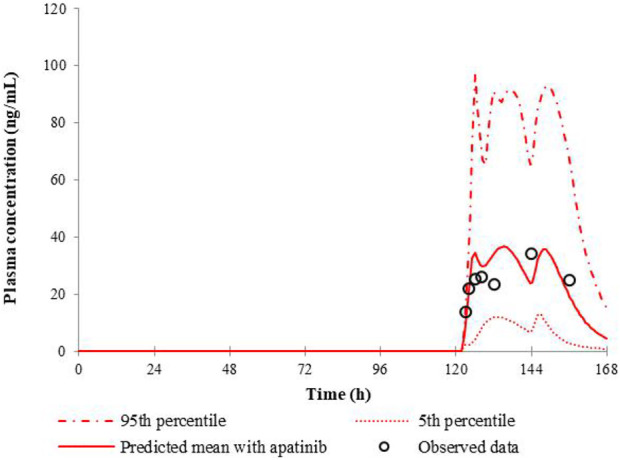
Predicted and observed mean plasma concentration-time curves of nifedipine following 30 mg nifedipine CR tablet in the presence of apatinib.

The predicted and the observed mean plasma concentration-time curves of nifedipine with apatinib in cancer patients are shown in Figure 2. Table 4 shows calculated AUC ratio as well as C_max_ ratio in the presence to the absence of apatinib. The successful simulation of the pharmacokinetic DDIs between nifedipine and apatinib is obtained by the established PBPK model. The increases of the predicted AUC and C_max_ of nifedipine in the presence of apatinib are 1.73 and 1.41, respectively.

**TABLE 4:** Comparison of model-predicted AUC, and C_max_ to the observed one in the presence to the absence of apatinib.

Administrated drugs	Simulated				Observed				C_max_ ratio_s_/C_max_ ratio_o_	AUC ratio_s_/AUC ratio_o_
	C_max_ (ng/ml)	C_max_ ratio	AUC (ng/mL·h)	AUC ratio	C_max_ (ng/ml)	C_max_ ratio	AUC (ng/mL·h)	AUC ratio		
Nifedipine	20.97		495.09		23.20		590.00			
		1.41		1.73		1.58		1.80	0.89	0.96
Nifedipine + apatinib	29.51		854.64		36.60		1060.00			

### Verification of pharmacodynamic model for nifedipine

**FIGURE 3:**
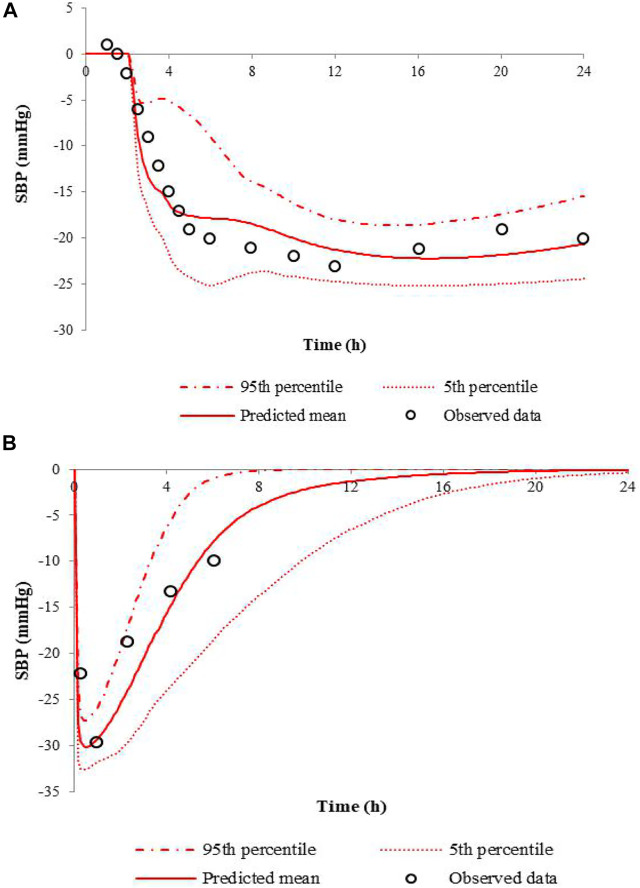
Predicted SBP changes and clinical observations in hypertensive patients at a single dose of 60 mg nifedipine CR tablet **(A)**, and 10 mg nifedipine IR tablet **(B)**.

When the E_max_ was set to −30 mmHg, the PD model fitted best. The comparison of the predicted SBP changes to the observations for nifedipine CR tablet at 60 mg or IR tablet at 10 mg dose levels is presented in Figure 3. Results show that the PD models for nifedipine CR tablet and IR tablet are successfully developed in predicting the clinical data. The predicted and observed values of R_max_ and AUE ratios are listed in Table 5. The ratios are all between 0.5 and 2-fold. The results indicate the good predictive performance of the current nifedipine PD model.

**TABLE 5:** Comparison of the predicted R_max_, and AUE to the observations for nifedipine CR tablet and IR tablet.

Dose level	Parameters	Unit	Predicted	Observed	Fold error
60 mg nifedipine CR tablet	R_max_	mmHg	−22.21	−23.09	0.96
	AUE	mmHg·h	433.98	440.10	0.99
10 mg nifedipine IR tablet	R_max_	mmHg	−30.13	−33.20	0.91
	AUE	mmHg·h	144.84	102.00	1.42

### Application of the physiologically-based pharmacokinetic/pharmacodynamic model for dosing recommendations

**FIGURE 4:**
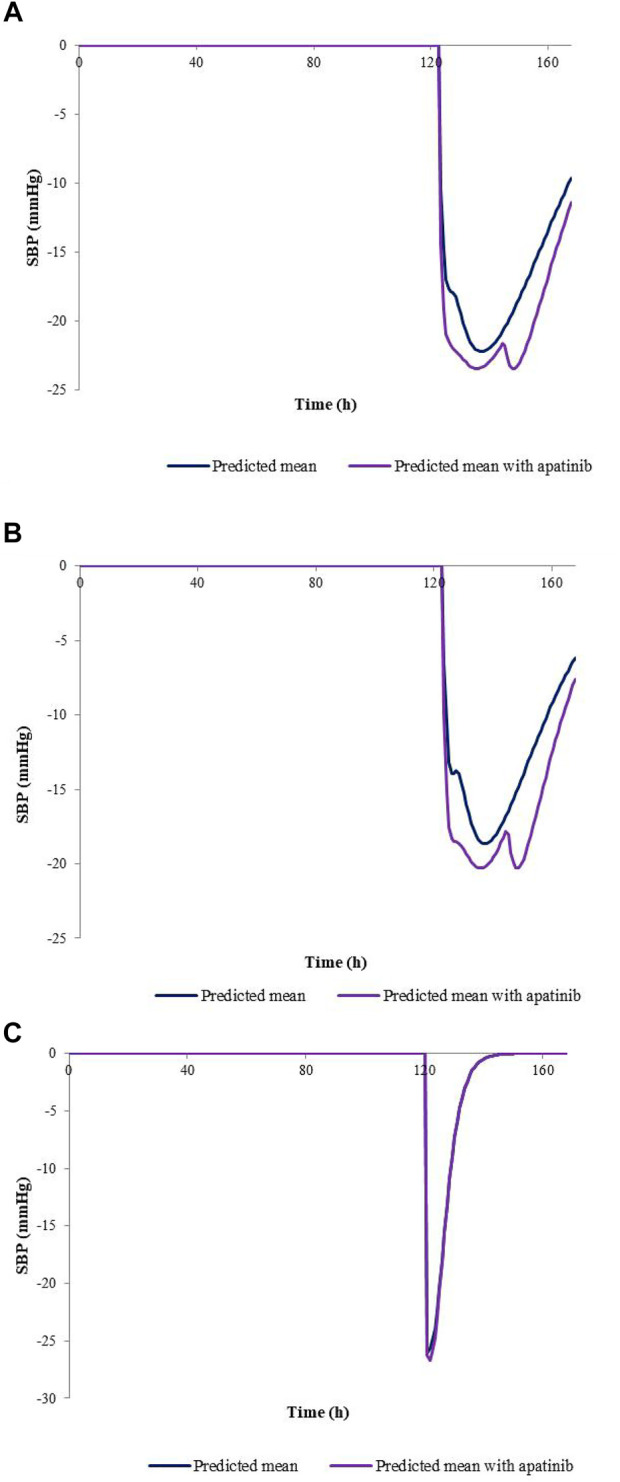
Predicted SBP changes for nifedipine CR tablet at 60 mg **(A)**, 30 mg **(B)** dose levels, and IR tablet at 30 mg **(C)** dose levels with or without co-administration of apatinib in cancer patients.

The developed PBPK/PD model was used to predict SBP changes caused by the exposure changes of nifedipine with the combination of apatinib in cancer patients. The model-predicted SBP changes for nifedipine CR tablet at 60 mg or 30 mg dose levels and IR tablet at 30 mg dose levels with or without co-administration of apatinib are presented in Figure 4. The calculated R_max_ and AUE ratio in the presence to the absence of apatinib in cancer patients are listed in Table 6. Table 7 summarizes the changes of SBP in hepatic impairments when combination of nifedipine with apatinib. The predicted R_max_ and AUE changes of nifedipine in the presence to the absence of apatinib in cancer patients and in hepatic impairments are all within 1.25-fold. Results show that the exposure changes of nifedipine caused by co-administration of apatinib has little effect on the SBP, indicating that apatinib is unlikely to cause severe pharmacodynamic DDI *via* inhibition of CYP3A4. Thus, nifedipine could be used in combination with apatinib without dose adjustment in clinic.

**TABLE 6:** Calculated R_max_ and AUE ratio in the presence to the absence of apatinib in cancer patients.

Dose level	Parameters	Unit	Nifedipine alone	Nifedipine combined with apatinib	Ratio
60 mg nifedipine CR tablet	R_max_	mmHg	−22.21	−23.44	1.06
	AUE	mmHg·h	787.55	916.08	1.16
30 mg nifedipine CR tablet	R_max_	mmHg	−18.62	−20.30	1.09
	AUE	mmHg·h	607.64	752.79	1.24
30 mg nifedipine IR tablet	R_max_	mmHg	−26.12	−26.67	1.02
	AUE	mmHg·h	206.78	210.03	1.02

**TABLE 7:** Calculated R_max_ and AUE ratio in the presence to the absence of apatinib in patients with hepatic impairments at a single dose of 60 mg nifedipine CR tablet.

Hepatic impairments	Parameters	Unit	Nifedipine alone	Nifedipine combined with apatinib	Ratio
Child-pugh A	R_max_	mmHg	−21.44	−21.64	1.01
	AUE	mmHg·h	749.26	775.87	1.04
Child-pugh B	R_max_	mmHg	−23.83	−24.18	1.01
	AUE	mmHg·h	909.16	949.93	1.04
Child-Pugh C	R_max_	mmHg	−24.97	−25.49	1.02
	AUE	mmHg·h	999.17	1055.30	1.06

## Discussion

Many cancer patients are also treated with antihypertensive agents concomitantly. Thus, the combination of CCB is unavoidable. Nifedipine is a commonly used antihypertensive drug in clinical practice and is also a sensitive substrate of CYP3A4. Besides, hypertension is a commonly reported adverse event for patients who receive apatinib, and nifedipine is most frequently used for drug-induced hypertension. Clinical study has revealed that the occurrence rate of hypertension as the adverse event of apatinib reached 73.9%, which necessitated the concurrent use of antihypertensive drugs (Zhu et al., 2020). Apatinib was reported to be a potent inhibitor of CYP3A4, with IC_50_ values of 1.80 and 0.83, and the corresponding K_i_ of 0.71 and 0.27 for midazolam hydroxylation and testosterone hydroxylation, respectively, indicating that apatinib might cause pharmacokinetic DDI *via* inhibition of CYP3A4 (Zhu et al., 2020). Therefore, it is of great significance to assess the magnitude of the DDI between nifedipine and apatinib. It has been shown that compared with the single oral administration of nifedipine, coadministration with apatinib contributed to the significant increases of AUC_0–48h_ and C_max_ of nifedipine by 83%, and 64%, respectively (Zhu et al., 2020). But whether this pharmacokinetic DDI between nifedipine and apatinib could enhance the antihypertensive effect of nifedipine, causing sever changes of blood pressure is unknown, which is critical for dosing regimens.

In the development of the DDI model between apatinib and nifedipine, the type of interaction and related enzyme inhibition parameters were crucial. The types of inhibition in Simcyp included competitive inhibition and mechanism-based inhibition (time-dependent inhibition), with the key parameters of K_i_ for competitive inhibition, and K_app_, as well as K_inact_ for mechanism-based inhibition, respectively. There were two studies regarding the kinetic parameters of enzyme inhibition by apatinib. The IC_50_ and IC_50_-shift results indicated that apatinib might not be a time-dependent inhibitor (Bao et al., 2018; Zhu et al., 2020). On the basis of the research, apatinib might inhibit CYP3A4 in a competitive way. However, it was found that the K_i_ values reported in these two articles were quite different. The K_i_ value of midazolam hydroxylation reported by Zhu et al. (2020) was 0.71 μM *via* a competitive inhibition, while a much higher K_i_ value of 11.5 μM was estimated in another study, also with midazolam as a probe substrate (Bao et al., 2018). Due to the fact that the experiment conducted by Zhu et al. was not published, we couldn’t compare the difference of the experiments between the two papers. We then used these two data as the model input parameters and conducted the DDI simulations separately. The simulation results were compared with the clinical observations. It was found that the predicted value was much closer to the clinical observation value in the competitive inhibition mode, with K_i_ of 0.71 μM as the initial value.

PK/PD modelling can elucidate the causative relationship between drug exposure and response, providing a better understanding of the pharmacological effects that results in the observed drug effect. In the simplest form, the measured plasma concentration serves as input for the concentration-effect relationship. And the observed pharmacological effects are directly linked to the site concentration. The classic and the most common PD model under these conditions is the E_max_ model. Many reports have repeatedly confirmed a close and direct relationship between circulating drug concentrations and antihypertensive effect (Abernethy et al., 1990; Donnelly et al., 1993; Donnelly et al., 1994; Meredith et al., 1994). Furthermore, it has been demonstrated that the magnitude of the first-dose responsiveness correlates with the responsiveness during steady-state treatment (Donnelly et al., 1993; Donnelly et al., 1994). This correlation creates the possibility of predicting the response to long-term treatment with nifedipine in individual patients (Meredith et al., 1994). Similar PKPD studies have demonstrated that the antihypertensive responses to other dihydropyridine CCBs, including amlodipine and felodipine, are directly related to the plasma concentration–time profiles for each drug (Edgar et al., 1987; Abernethy et al., 1990; Donnelly et al., 1993). In the present study, the PBPK model for nifedipine was linked to the PD model to predict the changes in blood pressure caused by the exposure changes of nifedipine with the combination of apatinib. The results might be helpful to the dosing regimen for combined administration. That is to say, when apatinib and nifedipine are co-administered, should the nifedipine be administered in regular dose in clinic, or the dose needs to be reduced. If the dose of nifedipine needs to be reduced, what is the appropriate dose?

The CR and IR formulations of nifedipine are most commonly used in clinic. In the study, the two formulations were both incorporated in the development of PBPK models for nifedipine. Owing to the fact that the absorption process of the CR tablet needs to be described by combining the *in vitro* dissolution data as well as the membrane permeability, the ADAM model was used for nifedipine CR tablet. The *in vivo* absorption of nifedipine IR tablet was described by the first-order model.

It has been reported that the control of SBP is more important than diastolic blood pressure (DBP) in most patients with hypertension (Levine et al., 2003; Mourad, 2008). Hence, SBP was employed in the PD modelling. An ordinary E_max_ model (Eq. 3) was used to describe the relationship between nifedipine concentration and the changes in SBP. The EC_50_ was reported to be 12.12 ng/ml for nifedipine at the regular doses (Hirasawa et al., 1985; Levine et al., 2003; Meredith and Elliott, 2004; Niu et al., 2021). So, the EC_50_ value was set to 12.12 ng/ml in this study. Based on the range of E_max_ values reported in the literature (Hirasawa et al., 1985; Levine et al., 2003; Meredith and Elliott, 2004; Niu et al., 2021), the PD model under different E_max_ values was examined to fit the observed SBP change caused by nifedipine at therapeutics doses. It has been found that the E_max_ was set to −30 mmHg, the PD model fitted best. The ratios of the predicted and observed values of R_max_ and AUE for nifedipine CR tablet as well as IR tablet were within 2, indicating the good predictive performance of the current nifedipine PD model.

Studies have shown that compared with the single oral administration of nifedipine, co-administration with apatinib contributed to the significant increases of AUC_0–48h_ and C_max_ of nifedipine by 83%, and 64%, respectively (Zhu et al., 2020). In the present study, similar statistical results are also seen based on the AUC and C_max_ increases of 1.73 and 1.41, respectively, after the co-administration of apatinib. The results suggest that coadministration of apatinib could significantly increase the exposure of nifedipine. But whether this pharmacokinetic DDI between nifedipine and apatinib could enhance the antihypertensive effect of nifedipine, causing sever changes of blood pressure is unknown, which is critical for dosing regimens. Hence, the developed PBPK/PD model was used to evaluate the changes in SBP caused by the exposure changes of nifedipine with the combination of apatinib. Two clinical scenarios were simulated using Simcyp virtual populations. The first scenario was designed to determine whether co-administration of apatinib with regular dose level of nifedipine could cause severe changes of SBP in cancer patients. The second scenario was to determine if dose adjustment of nifedipine was necessary in the combination of apatinib in patients with hepatic impairment. Our study shows that apatinib is unlikely to cause severe pharmacodynamic DDI *via* inhibition of CYP3A4. It is suggested that nifedipine could be used in combination with apatinib without dose adjustment in clinic. Further clinical studies in small sample are required to confirm the results.

However, there are some limitations in this study which warrant further discussion. First, due to lack of the observed data, the predictive performance of DDI for the nifedipine SR/CR formulations is not verified in this study. Second, although CYP3A4 might be more important in elimination of nifedipine compared to CYP3A5, few studies have suggested that CYP3A5 genotypes might explain variability in systemic exposure to nifedipine to certain extent (Haas et al., 2013). This was particularly important since the frequency of CYP3A5 expressors in Chinese population was highly relative to that in populations of European ancestry. Considering the inhibitory effect of apatinib on CYP3A4, we first conduct a DDI study on the CYP3A4. The effect of CYP3A5 is not yet evaluated. Third, a common adverse effect of apatinib was gastrointestinal disturbances, which might affect the transit time across different gastrointestinal segments, particularly for the controlled release formulation. Besides, nifedipine solubility might also be affected by variation in gastrointestinal pH. These are also not considered in the simulations. Despite the limitation existing in our refined model, we still believe that the model can give a rough understanding of the possible exposure change caused by DDI as well as the resulted pharmacodynamic changes to avoid some dangerous DDI in advance.

## Conclusion

In the present study, we have investigated the DDI between nifedipine and apatinib. Results show that apatinib could increase the exposure of nifedipine. But this exposure changes has little impact on the antihypertensive effect of nifedipine. Apatinib is unlikely to cause severe pharmacodynamic DDI *via* inhibition of CYP3A4. It is suggested that nifedipine could be used in combination with apatinib without dose adjustment in clinic.

## Data Availability

The original contributions presented in the study are included in the article/Supplementary Material, further inquiries can be directed to the corresponding authors.
